# Oxidative Stress and Neuroimaging Alterations in Major Depressive Disorder: A Systematic Review

**DOI:** 10.3390/ijms27177961

**Published:** 2026-09-07

**Authors:** Francisco Javier Gutiérrez-Corral, Ana Elizabeth González-Santiago, Alejandro Salvador Gómez-Cabrera, Rolando Castañeda-Arellano, Fernanda Isadora Corona-Meraz, María Guadalupe Sánchez-Parada

**Affiliations:** 1 Departamento de Ciencias Biomédicas, División de Ciencias de la Salud, Centro Universitario de Tonalá, Universidad de Guadalajara, Tonalá 45425, Jalisco, Mexico; 2 Departamento de Ciencias de la Salud-Enfermedad como Proceso Individual, División de Ciencias de la Salud, Centro Universitario de Tonalá, Universidad de Guadalajara, Tonalá 45425, Jalisco, Mexico; 3 Doctorado en Investigación Multidisciplinaria en Salud (DIMS), Centro Universitario de Tonalá, Universidad de Guadalajara, Tonalá 45425, Jalisco, Mexico

**Keywords:** major depressive disorder, oxidative stress, glutathione, neuroimaging, fMRI, magnetic resonance spectroscopy

## Abstract

Major depressive disorder (MDD) is associated with complex neurobiological alterations in which oxidative stress may play a central role; however, direct evidence linking redox biomarkers to alterations in brain chemistry remains limited. This systematic review aimed to synthesize the available evidence on the relationships between oxidative stress and neuroimaging alterations in individuals with MDD. A search was conducted in PubMed, Scopus, and Web of Science (2016–2026) following the PRISMA guidelines, including human studies assessing oxidative biomarkers (central or peripheral) alongside neuroimaging or neurofunctional measures. Four studies met the inclusion criteria (case–control, cross-sectional, and interventional designs). These studies collectively indicate that antioxidant enzymes, glutathione, and malondialdehyde are associated with altered brain activity and connectivity, prefrontal neurochemical changes, and cognitive performance. Overall, the findings support the existence of associations between redox disruption and central nervous system (CNS) alterations in MDD. However, methodological heterogeneity and the limited number of studies highlight the need for further longitudinal and multimodal research to clarify the directionality and clinical relevance of these findings.

## 1. Introduction

Major depressive disorder (MDD) is one of the leading psychiatric disorders worldwide. Its contribution to the global burden of mental disorders has grown over the past three decades, and it is presently recognized as a highly prevalent and disabling condition [1,2]. Its etiology is complex and multifactorial, arising from interactions between genetic, neurochemical, neuroendocrine, and environmental factors. MDD is considered one of the leading causes of disability worldwide and ranks prominently among mental disorders associated with suicide-related mortality [2]. Clinically, it is characterized by depressed mood, diminished interest in daily activities or pleasure, psychomotor agitation or retardation, insomnia or hypersomnia, feelings of worthlessness or excessive or inappropriate guilt, diminished ability to think or concentrate, recurrent suicidal ideation, significant weight loss or gain, decrease or increase in appetite, and fatigue or loss of energy. In MDD, these symptoms are not attributable to the physiological effects of a substance or another medical condition [3], and can significantly impair the affected individual’s quality of life by disrupting cognitive domains such as processing speed, learning and memory, perception, autobiographical memory, cognitive shifting, and intelligence quotient (IQ) [4]. According to the World Health Organization (WHO), depression is projected to become one of the leading contributors to the global burden of disease in the coming decades [1,2], underscoring its relevance as a major public health concern.

Beyond the classical models centered on monoaminergic neurotransmission, contemporary evidence supports an integrative view in which oxidative stress, neuroinflammation, and bioenergetic dysfunction substantially contribute to the pathophysiology of MDD [5].

Oxidative and nitrosative stress occur when the excessive production of reactive oxygen species (ROS) and reactive nitrogen species (RNS) is not balanced by endogenous antioxidant mechanisms, such as catalase, superoxide dismutase (SOD), glutathione peroxidase (GPx), and non-enzymatic antioxidants [6,7,8,9,10,11,12,13,14,15]. Mitochondrial activity generates superoxide, which is immediately converted to H_2_O_2_ by superoxide dismutase (SOD2) in the mitochondrial matrix and SOD1 in the cytosol. SOD1 and SOD2 protect against protein degradation by inactivating iron–sulfur protein clusters [7,9].

H_2_O_2_ can be converted to a highly reactive hydroxyl radical—the most harmful ROS—by means of the Fenton reaction [11]. Alternatively, H_2_O_2_ is converted to water by mitochondrial glutathione (GSH), together with GSH reductase and peroxiredoxins. GSH also plays a critical role in protecting lipids and defending cell membranes [10].

Reactive nitrogen species include the highly reactive peroxynitrite (ONOO^−^), produced by the reaction of superoxide and nitric oxide (NO). Through its reaction with CO_2_, ONOO^−^ also stimulates the production of the highly reactive radicals •NO_2_ (nitrogen dioxide) and CO_3_•^−^ (carbonate radical), which, together with NO, can additionally generate N_2_O_3_ (dinitrogen trioxide). NO produced by NO synthase can regulate physiological processes in neurons, including blood flow, cell differentiation, and neurotransmission [6,8].

Sustained exposure to ROS/RNS promotes lipid peroxidation, leading to impaired cellular function, loss of membrane elasticity and fluidity, and potentially cell death [15]. These processes may induce direct neuronal damage and disrupt neurotransmission and brain homeostasis; however, the specific mechanisms through which oxidative stress is linked to MDD are not yet fully understood [14,16].

In the presence of an active inflammatory process, ROS/RNS production increases, generating cellular and tissue damage through enhanced oxidative stress and inducing mitochondrial alterations [11,12,16]. Due to their central role in oxidative phosphorylation, mitochondria represent a major source of free radicals, and the electron transport chain itself is particularly vulnerable to oxidative damage [11,15,16]. Such alterations have been described in several mental disorders, including MDD and anxiety disorders [15,17,18].

In this context, MDD has been associated with the concept of “accelerated aging,” in which decreased leukocyte telomere length has been reported to be associated with lifetime exposure to MDD [16,18]. Additionally, sustained increases in oxidative stress, when coexisting with chronic conditions such as cardiovascular diseases, diabetes, dementia, and osteoporosis, may amplify the systemic inflammatory status [19,20,21,22]. Furthermore, sociodemographic and lifestyle factors such as smoking, alcohol consumption, and physical inactivity may predispose individuals to greater neuronal damage [23]. Reciprocally, MDD increases the risk of developing cardiovascular disease [24], cancer [25], Alzheimer’s disease [26], obesity [27], and diabetes [28], which may contribute to a self-perpetuating cycle of inflammation and oxidative stress [12,29].

Several studies have reported a higher incidence of vascular alterations in patients with MDD, which has been associated with endothelial dysfunction assessed via flow-mediated dilation (FMD) [30]. These alterations suggest reduced bioavailability of vascular function associated with nitric oxide (NO), although the underlying molecular mechanisms have not yet been fully characterized [30,31].

Numerous investigations have addressed the pathophysiological mechanisms of depression [21]. The main hypotheses focus on alterations in monoaminergic neurotransmission, dysfunction of the hypothalamic–pituitary–adrenal (HPA) axis [32], and neuroinflammation [33], with growing evidence also supporting the role of oxidative stress in the pathogenesis of MDD [5,14]. In particular, the hippocampus—a key structure for memory and cognitive processes—is especially vulnerable to oxidative damage, which may lead to reduced levels of essential molecules such as brain-derived neurotrophic factor (BDNF) [4,34,35], suggesting a potential pathological pathway between oxidative stress and the dysfunction of neuronal circuits, which are critical for cognition and emotional regulation [4,34,35].

Various biomarkers have been proposed for the assessment of oxidative stress in patients with MDD, including 8-hydroxy-2-deoxyguanosine (8-OHdG) and F2-isoprostanes; however, due to heterogeneity in the reported findings, none has proven to be a definitive marker [14]. Clinical studies have shown that patients with MDD exhibit higher levels of oxidative stress compared with healthy subjects, and that antidepressant treatments may modify certain biomarkers such as isoprostanes, reinforcing the need for a comprehensive evaluation of redox status [12,15,17,35,36,37,38].

Tissue changes potentially related to oxidative stress mechanisms in patients with MDD may be accompanied by cognitive and memory impairment. Neuroimaging studies have identified reductions in prefrontal cortex and hippocampal volume [36], contributing to the understanding of executive and memory dysfunction.

Furthermore, patients with MDD exhibit neurochemical differences in the brain related to HPA axis activity, disorder severity, and cognitive function. Reduced hippocampal volume in patients with MDD has been explained through two putative mechanisms, namely, neuroplasticity and neurogenesis [37]. Hippocampal volume alterations may help to explain the latency of response to antidepressant treatments, which share a common effect in modulating neuroplasticity and influencing depression severity [38]. This reinforces the need for integrative approaches that consider molecular mechanisms and neuroimaging findings alongside the clinical features of MDD [39].

Nevertheless, the molecular mechanisms linking oxidative stress to alterations in brain chemistry and to the clinical manifestations of MDD remain incompletely elucidated [14].

**Figure 1:**
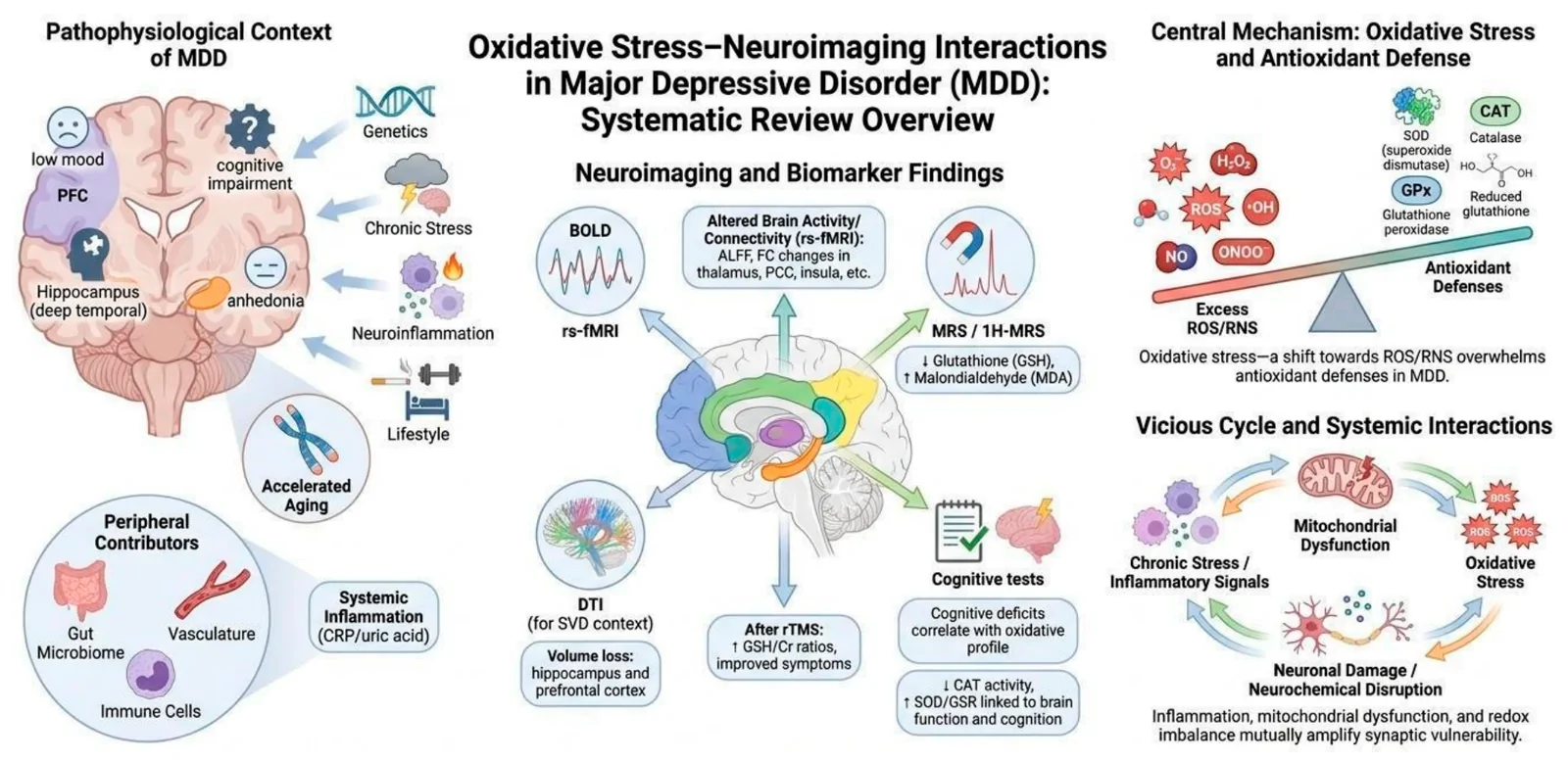
Oxidative stress and neuroimaging interactions in Major Depressive Disorder (MDD). Systemic inflammation and lifestyle factors may contribute to oxidative stress and neural changes in MDD. Risk factors such as chronic stress, poor lifestyle, and aging trigger systemic inflammation, mediated by gut, immune, and vascular interactions. This inflammation correlates with key depressive symptoms such as low mood, cognitive impairment, and anhedonia associated with regions including the prefrontal cortex and hippocampus. Neuroimaging reveals how this stress impacts the brain: resting-state fMRI shows altered connectivity in areas such as the thalamus and insula, DTI highlights volume loss, and proton magnetic resonance spectroscopy (^1^H-MR) indicates reduced glutathione (GSH) alongside elevated malondialdehyde (MDA) levels. At the molecular level, excessive reactive oxygen and nitrogen species (ROS/RNS) overwhelm the body’s antioxidant defenses (SOD, CAT, GPx). This is proposed to initiate a self-amplifying cycle in which chronic inflammation, mitochondrial dysfunction, ROS accumulation, and neuronal damage may reinforce one another, potentially contributing to synaptic vulnerability and cognitive decline. Abbreviations: MDD, Major Depressive Disorder; PFC, prefrontal cortex; ROS/RNS, reactive oxygen/nitrogen species; SOD, superoxide dismutase; CAT, catalase; GPx, glutathione peroxidase; GSH, reduced glutathione; MDA, malondialdehyde; rs-fMRI, resting-state functional magnetic resonance imaging; ALFF, amplitude of low-frequency fluctuations; FC, functional connectivity; PCC, posterior cingulate cortex; MRS/1H-MRS, proton magnetic resonance spectroscopy; DTI, diffusion tensor imaging; SVD, small vessel disease; rTMS, repetitive transcranial magnetic stimulation; CRP, C-reactive protein. Arrows indicate proposed directional or bidirectional relationships among pathophysiological factors, oxidative stress, neuroinflammation, mitochondrial dysfunction, neuronal damage, and neuroimaging findings; they represent conceptual associations rather than demonstrated causal relationships.

Overall, the evidence presented to date suggests that oxidative stress, mitochondrial dysfunction, and alterations in neuroendocrine and inflammatory pathways may all play important roles in the pathophysiology of MDD. The proposed interactions among these pathophysiological mechanisms and their relationship with neuroimaging and biomarker findings are summarized in Figure 1. Therefore, this systematic review aims to synthesize the available literature linking oxidative stress with neuroimaging alterations in MDD, with emphasis on redox biomarkers, their interactions with the HPA axis, and inflammation.

## 2. Methods

The search protocol for this systematic review was designed in accordance with the Preferred Reporting Items for Systematic Reviews and Meta-Analyses (PRISMA) [40] guidelines, ensuring a transparent and comprehensive search of recent and relevant studies on the research topic. The PRISMA checklist is available at the Supplementary Materials Section.

### 2.1. Study Design

A systematic review was performed to identify studies on oxidative stress, neuroimaging, and brain chemistry alterations in the context of major depressive disorder. The search was conducted in the PubMed, Scopus, and Web of Science electronic databases.

### 2.2. Eligibility Criteria

A search strategy was developed to collect the most relevant studies investigating the relationships between oxidative stress and brain damage in MDD. The search was limited to English-language studies published between 1 January 2016 and 1 January 2026, due to resource and time constraints. Randomized trials and observational studies (cohort, case–control, and cross-sectional designs) were included. Eligible outcomes were required to link oxidative stress biomarkers (measured centrally or via peripheral sampling) to alterations in brain chemistry and to neuroimaging or neurofunctional measures in patients with MDD. Studies not conducted on humans, case reports, editorials, letters to the editor, and studies without full-text availability were excluded. Additionally, articles from journals not indexed in the Journal Citation Report (JCR) were excluded. Although Web of Science provides access to journals indexed in JCR, PubMed and Scopus were incorporated to increase the sensitivity of the search and broaden its coverage. Subsequently, potentially relevant studies were evaluated according to their JCR indexing status. Additional eligibility criteria were as follows: studies conducted on humans, subjects aged >18 years, a diagnosis of MDD according to the DSM-5-TR or ICD-10, oxidative stress biomarkers, and neuroimaging or neurofunctional assessment.

#### Literature Search and Selection of Articles

The search was conducted in the PubMed, Scopus, and Web of Science databases (accessed 5 January 2026). Combinations of keywords and Medical Subject Headings (MeSHs) related to major depressive disorder, oxidative stress, and brain injury were used. The Boolean operators “AND” and “OR” were used to combine search terms effectively. The search strategy included combinations and variations of the following terms: “major depressive disorder” AND “oxidative stress”, “brain chemistry alterations” AND “major depression” AND “free radicals” AND “brain injury by neuroimaging”; “MDD” AND “redox biomarkers”; “major depressive disorder” AND “oxidative stress” AND “brain injury”; “major depression” AND “free radicals” AND “brain injury by neuroimaging”; “MDD” AND “redox biomarkers”; “brain injury” AND “neuroimaging” AND “association”; “major depression” AND “inflammation-related damage” AND “relationship with oxidative stress”; “MDD” AND “inflammatory biomarkers” AND “brain alterations”; “neuroimaging.” In addition, a manual review of the reference lists of the most relevant articles identified during the initial search was performed to detect additional studies that may not have been captured.

**Figure 2:**
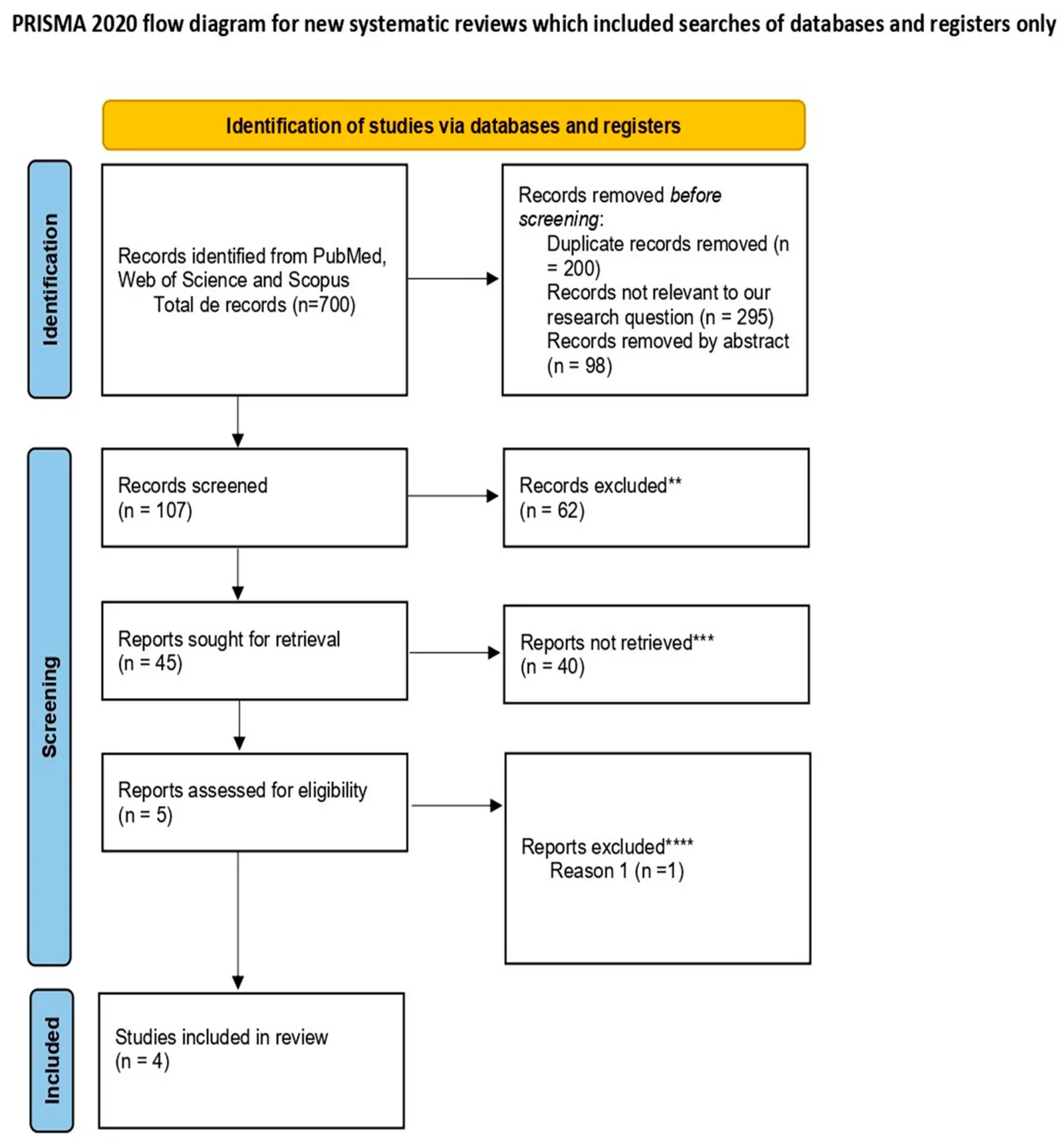
Study identification according to the PRISMA 2020 guidelines. ** Studies not relevant to the present review. *** The full text could not be retrieved. **** Reason 1: Animal study.

All search results were recorded, and duplicate studies were removed. Retrieved studies underwent a structured selection process based on predefined inclusion and exclusion criteria to ensure the relevance and quality of each study included in the systematic review. The selection process is shown in Figure 2.

### 2.3. Data Extraction

Four articles met the inclusion criteria and were selected for data extraction. The following data were extracted: title, study design, population characteristics (ethnicity, age, sample size), measurement of biomarkers or agents related to oxidative stress, neuroimaging or neurofunctional measures (including directionality and associations), and relevant findings.

### 2.4. Risk of Bias Assessment Across Studies

The evaluation of the studies was performed by three authors, who independently assessed each study. The research team discussed any significant discrepancies until consensus was reached. Methodological quality was assessed using the Newcastle–Ottawa Scale (NOS) [41,42], which evaluates three domains: selection (four items), comparability (two items), and outcome (three items). According to the NOS criteria, studies scoring between 7 and 10 are considered high quality with low risk of bias; those scoring between 3 and 6 are considered moderate quality with moderate risk of bias; and those with lower scores are considered low quality with high risk of bias. For this review, studies with a score of 6 or greater were considered to have high methodological quality and are reported accordingly as having a low risk of bias. The pre–post interventional study design [43] was evaluated using the NHLBI (National Heart, Lung and Blood Institute, National Institutes of Health, Bethesda, MD, USA) Study Quality Assessment tool for pre–post studies [44].

In total, three studies used case–control designs, while one had a clinical pre–post interventional study design. Total scores ranged from 5 to 7, and the three case–control studies were classified as having low risk of bias (≥6) [41]. The remaining study was evaluated using the NHLBI Study Quality Assessment tool for pre–post studies [44]. This tool cannot be considered standardized and is considered a tool that assists in the detection of potential flaws in the methods or implementation of a study. In particular, the NHLBI assessment tool considers the study question and design, eligibility/selection criteria, sample size, consistency of test/service/intervention, valid outcome measures, blinded exposures/interventions, lost-to-follow-up accounts, and statistical tests [44]. The pre–post interventional study [43] had one flaw in its eligibility/selection criteria, had no blinded intervention, and was not an interrupted time-series design. Overall, the three case–control studies demonstrated adequate case definition, objective outcome measurement, and appropriate statistical analyses; however, in some cases, formal sample size calculation and detailed reporting of non-response rates were not provided. These findings suggest that the included studies are characterized by overall moderate-to-high methodological quality.

### 2.5. Synthesis of Results

Due to heterogeneity in study design, biomarkers, and neuroimaging/neurofunctional assessments, a meta-analysis was not performed. Instead, a narrative synthesis was conducted, grouping results according to the type of oxidative stress biomarker and neuroimaging/neurofunctional modality, while considering population differences and treatments that could explain any discrepancies.

## 3. Results

A total of 700 records were identified through searches in PubMed, Scopus, and Web of Science. After removal of 200 duplicate records, a further 295 and 98 studies were excluded as their titles and abstracts, respectively, indicated that they were not relevant to our research question. A total of 107 unique references remained after title and abstract screening. Of these, 45 were selected for full-text evaluation. Subsequently, 41 articles were excluded due to failure to meet the predefined eligibility criteria. Finally, four studies met all criteria and were included in the synthesis. The final selected studies assessing the associations between oxidative stress biomarkers and central nervous system alterations in major depressive disorder (MDD) are summarized in Table 1.

### 3.1. General Characteristics of the Included Studies

The four included studies were heterogeneous in design and outcomes, detailed as follows: (1) one case–control study using resting-state functional magnetic resonance imaging (rs-fMRI) in first-episode, drug-naïve MDD, evaluating plasma antioxidant biomarkers and their associations with brain activity/connectivity [45]; (2) one case–control study integrating spectroscopy (MRS) and rs-fMRI to quantify cerebral glutathione and its relationship with spontaneous neuronal activity [47]; (3) one pre–post interventional study evaluating repetitive transcranial magnetic stimulation (rTMS), with changes in prefrontal metabolites measured via proton magnetic resonance spectroscopy (^1^H-MRS) [43]; and (4) one case–control study assessing peripheral oxidative stress markers and cognitive performance, including mediation analyses according to symptom severity [46].

Collectively, the four included studies evaluated complementary biomarkers of oxidative stress, including antioxidant enzymes (SOD, GSR, and catalase), glutathione-related metabolites (GSH), and lipid peroxidation markers such as malondialdehyde (MDA), allowing for the assessment of different components of redox homeostasis in patients with major depressive disorder [43,45,46,47].

#### 3.1.1. Peripheral Oxidative Stress and Brain Connectivity/Activity in First-Episode MDD

In a cohort study using rs-fMRI in first-episode, drug-naïve MDD, including 57 patients (aged 18–55 years) with MDD and 60 controls (matched for age and sex), plasma levels of SOD and glutathione reductase (GSR) were measured. In the MDD group, associations were observed between SOD and GSR levels and alterations in spontaneous activity—measured via the amplitude of low-frequency fluctuation (ALFF)—in key regions (including the thalamus, anterior cingulate, and superior frontal gyrus), as well as associations with thalamus–insula functional connectivity (negative for GSR) and thalamus–precuneus connectivity (positive for SOD). Excessive oxidative stress was associated with alterations in neuronal activity, with different patterns between MDD subtypes. The study also reported differences in these associations according to the presence or absence of suicidal ideation, suggesting pathophysiological heterogeneity within MDD [45].

#### 3.1.2. Cerebral Glutathione (MRS) and Spontaneous Neuronal Activity

In a case–control study combining MRS (MEGA-PRESS sequence) and rs-fMRI, GSH and glutamate were quantified in the medial prefrontal cortex and precuneus, and comparisons were made between healthy controls (*n* = 30), patients with MDD (*n* = 28), and patients with OCD (*n* = 28). Lower GSH concentrations in the medial prefrontal cortex and precuneus were reported in MDD and OCD patients compared with controls; although associations between GSH levels and spontaneous neuronal activity metrics were explored, no association with symptoms was found [47].

#### 3.1.3. rTMS and Prefrontal Neurochemical Changes Measured via 1H-MRS

In a pre–post interventional study in patients with MDD (*n* = 18), the efficacy of repetitive transcranial magnetic stimulation (rTMS) and changes in prefrontal metabolites measured via ^1^H-MRS were evaluated. A significant reduction in depression scores—measured using the Hamilton Depression Rating Scale (HAMD)—was reported following rTMS, along with significant increases in metabolite ratios (NAA/Cr, GSH/Cr, and Gln/Cr) after treatment, suggesting possible changes in the neurochemical microenvironment associated with redox balance and glutamatergic neurotransmission [43].

#### 3.1.4. Peripheral Oxidative Stress and Neurocognitive Function in MDD

In a case–control study including 44 patients with MDD and 47 controls, plasma levels of malondialdehyde (MDA) and catalase (CAT) activity were measured, alongside cognitive assessments (RBANS and Stroop). The MDD group showed higher MDA levels and lower CAT activity, as well as significant deficits across multiple cognitive domains (immediate memory, attention, delayed memory, and total score). Correlations were reported between higher MDA levels and poorer mnemonic performance, as well as associations between MDA and the severity of depressive and anxious symptoms. The results of mediation analyses suggested that depression/anxiety severity may partially mediate the relationship between peripheral oxidative stress and cognitive performance [46].

## 4. Discussion

This systematic review synthesized the available evidence on the relationships between oxidative stress biomarkers and neuroimaging or neurofunctional outcomes in major depressive disorder (MDD). Although only four studies met the predefined eligibility criteria, their findings converged on the associations between redox dysregulation and abnormalities in functional brain activity, cerebral neurochemistry, cognitive performance and, in patients with a vascular depressive phenotype, structural brain connectivity [43,45,46,47,48]. Collectively, these findings reinforce the biological relevance of oxidative stress within the multifactorial pathophysiology of depression. Nevertheless, because the available evidence remains limited and is derived predominantly from observational studies, the reported associations should not be interpreted as evidence that oxidative stress directly causes the central nervous system alterations observed in MDD.

A key finding of this systematic review is that oxidative stress cannot be adequately characterized by a single biomarker. The included studies assessed distinct components of redox homeostasis, including superoxide dismutase (SOD), glutathione reductase (GSR), glutathione (GSH), catalase (CAT), malondialdehyde (MDA), and serum uric acid (SUA). SOD, GSR, CAT, and GSH primarily reflect endogenous antioxidant defense mechanisms, whereas MDA is a by-product of lipid peroxidation and therefore serves as a marker of oxidative damage [43,45,46,47]. Although SUA exhibits antioxidant activity in plasma, its concentration is also influenced by metabolic, renal, dietary, and pharmacological factors [48]. Consequently, these biomarkers should be considered complementary, rather than as interchangeable indicators of redox status. Previous systematic reviews and meta-analyses have similarly documented increased oxidative damage together with impaired antioxidant defenses in patients with depressive disorders while emphasizing substantial heterogeneity related to biological matrices, analytical methodologies, medication exposure, disease stage, and comorbidities [14,49]. These secondary findings provide broader biological context for the primary evidence included in the present review but should not be considered equivalent to direct biomarker–neuroimaging evidence.

The study by Liu et al. provided evidence that peripheral antioxidant biomarkers are associated with functional brain abnormalities in patients with first-episode, drug-naïve MDD. Plasma SOD and GSR concentrations were associated with altered spontaneous neuronal activity in the thalamus, anterior cingulate cortex, and superior frontal gyrus, as well as with disrupted functional connectivity between the thalamus and both the insula and precuneus [45]. These regions play central roles in emotional regulation, salience processing, interoceptive awareness, executive functioning, and self-referential cognition, all of which have frequently been reported to be disrupted in patients with depressive disorders. The observed association between peripheral antioxidant enzymes and these functional networks suggests that systemic redox dysregulation may reflect central neurobiological alterations rather than representing an isolated peripheral phenomenon. Furthermore, the distinct association patterns observed according to suicidal ideation support the biological heterogeneity of MDD and raise the possibility that redox alterations are linked to specific clinical and neurofunctional phenotypes [45]. However, due to the cross-sectional design, it remains unclear whether oxidative dysregulation precedes these functional abnormalities, results from them, or reflects a shared underlying pathological process affecting both.

Evidence derived from proton magnetic resonance spectroscopy (^1^H-MRS) provides a more direct assessment of cerebral antioxidant status. Lee et al. reported significantly lower GSH concentrations in the medial prefrontal cortex and precuneus of patients with MDD than in healthy controls when simultaneously evaluating spontaneous neuronal activity using resting-state functional magnetic resonance imaging [47]. As the principal intracellular antioxidant, GSH plays a pivotal role in peroxide detoxification, preservation of membrane integrity, and maintenance of neuronal redox homeostasis. Accordingly, reduced cerebral GSH concentrations may indicate diminished antioxidant capacity within brain regions critically involved in emotional regulation, cognitive control, and internally directed information processing. Notably, cerebral GSH levels were not significantly associated with symptom severity, suggesting that reduced brain GSH may represent a neurobiological characteristic of the disorder rather than a direct indicator of current clinical severity [47]. This interpretation is consistent with previous systematic reviews and meta-analyses of ^1^H-MRS studies reporting altered cerebral glutathione concentrations in patients with depression, although the limited number of available studies and methodological variability in image acquisition and metabolite quantification remain important considerations [50].

The pre–post rTMS study provides a preliminary longitudinal perspective. Following repetitive transcranial magnetic stimulation, patients exhibited significant reductions in Hamilton Depression Rating Scale scores together with increases in the prefrontal NAA/Cr, GSH/Cr, and Gln/Cr ratios [43]. The increase in GSH/Cr suggests that clinical improvement may be accompanied by changes in antioxidant-related cerebral metabolism, whereas alterations in Gln/Cr and NAA/Cr may reflect changes in glutamatergic cycling and neuronal metabolic integrity. These findings are particularly relevant because they indicate that redox-related metabolites are dynamic rather than static, and their levels may change during effective neuromodulatory treatment. However, this study included only 18 participants, lacked sham-treated and untreated control groups, and quantified metabolite ratios rather than absolute metabolite concentrations. As a result, it cannot be determined whether these metabolic changes mediated the antidepressant response, were a result of clinical improvement, or reflected nonspecific treatment- or time-related effects. Therefore, cerebral GSH should currently be regarded as a promising candidate biomarker of treatment response, rather than a clinically validated biomarker [43].

The study evaluating MDA, CAT, and cognitive performance provides an important clinical bridge between peripheral oxidative status and neuropsychological dysfunction [46]. Patients with MDD exhibited higher MDA concentrations, lower CAT activity, and poorer performance regarding immediate memory, attention, delayed memory, and global cognitive function [46]. Higher MDA levels were also associated with poorer memory performance and greater severity of depressive and anxiety symptoms. Furthermore, the results of mediation analyses suggested that the severity of depression and anxiety symptoms may partially mediate the relationship between oxidative stress and cognitive performance [46]. These findings indicate that an unfavorable peripheral redox profile is accompanied by cognitive dysfunction in MDD, particularly in memory-related domains. However, the mediation analysis results should not be interpreted as evidence of a causal biological pathway because the study employed a cross-sectional design. Oxidative damage, cognitive impairment, and symptom severity may influence one another bidirectionally or arise from shared mechanisms, including chronic stress, sleep disturbances, metabolic dysfunction, systemic inflammation, or medication exposure.

Collectively, the available evidence indicates that redox dysregulation is associated with abnormalities across multiple levels of central nervous system organization. Peripheral antioxidant enzymes were associated with alterations in resting-state brain activity and functional connectivity [45]; cerebral glutathione concentrations were reduced in the medial prefrontal cortex and precuneus [47]; neuromodulatory treatment was accompanied by changes in glutathione- and glutamine-related metabolites [43]; and peripheral oxidative damage was consistently associated with cognitive impairment [46]. Rather than supporting a single oxidative mechanism underlying MDD, these converging observations favor a multidimensional model in which oxidative stress interacts with mitochondrial function, inflammatory signaling, glutamatergic neurotransmission, neuroplasticity, and stress response pathways.

Inflammation represents a particularly important component of this framework because inflammatory activation promotes the generation of reactive oxygen species (ROS) and reactive nitrogen species (RNS), causing oxidative and nitrosative stress, which further amplifies inflammatory signaling and cellular injury [12,16,33]. Meta-analyses have consistently reported low-grade inflammatory activity—including elevated C-reactive protein concentrations—in subsets of patients with depression, and have reported the potential antidepressant effects of anti-inflammatory interventions in selected populations [29,33,51,52]. Collectively, these findings provide mechanistic support for an integrated inflammation–redox model of depression. That said, inflammatory biomarkers were not simultaneously assessed alongside neuroimaging outcomes in the studies included in this review. Accordingly, the evidence regarding inflammation should be interpreted as contextual and supporting a potential mechanistic interaction, rather than a direct causal conclusion derived from the present synthesis [53].

The relationships between redox dysregulation and neuronal dysfunction may also involve disturbances in glutamatergic metabolism, mitochondrial bioenergetics, membrane lipid peroxidation, and impaired neuroplasticity. Excessive production of ROS and RNS can damage lipids, proteins, and nucleic acids, whereas inadequate antioxidant defenses increase neuronal susceptibility to metabolic and inflammatory stresses [5,14,16]. Glutathione is particularly relevant because it contributes not only to antioxidant protection but also to glutamate metabolism. Therefore, the MRS findings involving GSH and glutamine are biologically consistent with an interaction between redox homeostasis and glutamatergic neurotransmission [43,47]. However, none of the available studies directly evaluated mitochondrial function, synaptic plasticity, brain-derived neurotrophic factor (BDNF) signaling, or oxidative modification of neuronal proteins. Therefore, these mechanisms should be regarded as biologically plausible interpretations rather than experimentally demonstrated pathways.

Serum uric acid (SUA) warrants particularly cautious interpretation. Previous meta-analyses have reported lower SUA concentrations in patients with MDD together with potential changes following antidepressant treatment, although the findings remain heterogeneous [54]. In the study discussed in the present review, lower SUA levels were associated with alterations in structural network organization and a greater burden of depressive symptoms among patients with cerebral small vessel disease [48]. These findings suggest a potential relationship between systemic antioxidant status and structural connectome vulnerability in the context of a vascular depressive phenotype. However, the study population was characterized by cerebral small vessel disease accompanied by depressive symptoms, rather than primary MDD alone. Furthermore, vascular pathology, aging, renal function, metabolic status, dietary habits, and medication use may all influence SUA concentrations. Accordingly, these findings should be viewed as complementary and hypothesis-generating, rather than as evidence supporting SUA as a specific biomarker of primary MDD or justifying its extrapolation to patients without cerebrovascular disease [48,53,55].

At present, the translational applicability of the biomarkers reviewed remains limited. None has accumulated sufficient evidence to serve independently as a diagnostic, prognostic, or treatment-selection biomarker. Peripheral biomarkers such as MDA, CAT, SOD, and GSR are readily accessible and relatively inexpensive; however, they are influenced by numerous systemic factors, including diet, smoking, physical activity, body mass index, inflammatory status, and medication use [14,17,18,45,49,56]. In contrast, cerebral glutathione quantified via proton magnetic resonance spectroscopy offers greater neurobiological specificity but requires specialized acquisition protocols, standardized voxel placement, advanced spectral processing, and rigorous control of scanner-related variability [43,47,50]; thus, its widespread clinical application remains limited due to cost and availability considerations. As a result, the most promising translational strategy is unlikely to rely on a single oxidative biomarker, but rather on multimodal biological profiles integrating peripheral redox biomarkers, cerebral metabolites, neuroimaging-derived characteristics, cognitive performance, inflammatory status, and detailed clinical phenotyping.

Future investigations should prioritize adequately powered longitudinal and multimodal study designs. Simultaneous assessment of peripheral and central redox biomarkers would help to determine whether systemic oxidative markers accurately reflect cerebral antioxidant status. Repeated evaluations before and after pharmacological, psychotherapeutic, or neuromodulatory interventions may clarify whether alterations in redox homeostasis precede, accompany, or follow clinical improvement. Future studies should also develop standardized biological sample collection and storage approaches, analytical platforms, MRS acquisition protocols, neuroimaging preprocessing pipelines, and clinical definitions. Particular attention should be paid to medication exposure, as antidepressants and other psychotropic agents may substantially influence oxidative and inflammatory biomarkers. Drug-naïve first-episode cohorts remain especially valuable for minimizing this important source of confounding, whereas chronic and treatment-resistant populations are essential for investigating disease progression and treatment-related biological changes. Finally, stratification according to suicidal behavior, anxiety severity, cognitive impairment, metabolic comorbidity, and inflammatory status may facilitate the identification of biologically meaningful subgroups within the heterogeneous clinical spectrum of MDD.

### Limitations and Methodological Considerations

This systematic review was conducted in accordance with the PRISMA 2020 guidelines [40]. Only human studies indexed in major scientific databases (PubMed, Scopus, and Web of Science) were included. Methodological quality was assessed using the Newcastle–Ottawa Scale (NOS) [41] for observational studies and the NHLBI Study Quality Assessment Tool for Before–After (Pre–Post) Studies [44]. Overall, the included studies were classified as having a low to moderate risk of bias, supporting the internal consistency and methodological robustness of the synthesized evidence.

Despite a comprehensive literature search, direct evidence linking oxidative stress biomarkers with central nervous system outcomes in humans remains limited. Studies simultaneously evaluating oxidative stress biomarkers and neuroimaging, proton magnetic resonance spectroscopy (^1^H-MRS), or other neurofunctional measures remain scarce, highlighting an important gap in the current literature [43,45,46,47].

Furthermore, considerable heterogeneity was observed across the included studies with respect to study design, sample characteristics, clinical stage, biomarker selection, biological matrices, neuroimaging modalities, cognitive assessment methods, and analytical approaches [43,45,46,47]. This methodological variability precluded quantitative meta-analysis and limited direct comparison between studies. In addition, most of the available evidence was cross-sectional, whereas the only interventional study employed a small, uncontrolled pre–post design [43]. Therefore, the evidence available at present does not allow conclusions regarding the temporal or causal relationships between oxidative stress and central nervous system abnormalities in MDD to be drawn.

Several potentially important confounding factors—including dietary habits, smoking status, physical activity, body mass index, sleep quality, baseline inflammatory status, medical comorbidities, and psychotropic medication use—were not consistently controlled across the included studies. Moreover, peripheral oxidative biomarkers are susceptible to preanalytical handling and laboratory-related variability, whereas MRS-derived metabolites and functional connectivity measures are influenced by image acquisition parameters, scanner characteristics, preprocessing pipelines, and analytical methodologies. These sources of variability should be carefully considered when interpreting the available evidence and when comparing findings across studies.

Finally, the evidence regarding serum uric acid (SUA) was derived from patients with cerebral small vessel disease, rather than individuals with primary MDD [48]. Accordingly, these findings should be interpreted as complementary evidence supporting the relevance of redox dysregulation in vascular depressive phenotypes rather than as direct evidence applicable to primary MDD.

Despite these limitations, the convergence of findings across studies utilizing peripheral oxidative biomarkers, resting-state functional MRI, ^1^H-MRS, treatment-related metabolic changes, and neuropsychological outcomes provides consistent support for the continued investigation of redox–brain interactions in patients with depression.

## 5. Conclusions

The available evidence links disrupted redox homeostasis to central nervous system abnormalities in individuals with major depressive disorder (MDD). In the retrieved articles, peripheral antioxidant enzymes were correlated with alterations in resting-state brain activity and functional connectivity, reduced prefrontal cortex and precuneal glutathione (GSH) concentrations were identified, clinical improvement following repetitive transcranial magnetic stimulation (rTMS) was accompanied by changes in prefrontal metabolites related to redox regulation and neurotransmission, and an unfavorable MDA/CAT profile was associated with cognitive impairment [43,45,46,47]. Furthermore, serum uric acid (SUA) findings point to redox–brain interactions in vascular depressive phenotypes, although this result cannot necessarily be generalized to primary MDD [48].

At the level of secondary evidence, systematic reviews and meta-analyses consistently indicate that increased oxidative stress together with impaired antioxidant defenses may represent a recurrent feature of depressive disorders, despite substantial methodological heterogeneity across studies [14,17,43,45,46,47,49,50,57]. Likewise, meta-analytic evidence supports the presence of low-grade inflammation—reflected by elevated C-reactive protein concentrations—as well as the potential antidepressant effects of anti-inflammatory interventions in selected patient subgroups, thereby reinforcing the integrative inflammation–redox–brain model [19,51,52].

Future longitudinal, adequately powered, and multimodal studies integrating peripheral and central redox biomarkers with inflammatory pathways, hypothalamic–pituitary–adrenal (HPA) axis function, and advanced neuroimaging techniques are warranted to clarify causal relationships, identify biologically meaningful subtypes of MDD, and determine whether restoration of redox homeostasis translates into sustained clinical improvement and cognitive recovery. Moreover, the converging evidence suggests that adjunctive therapeutic strategies targeting oxidative stress and inflammation may represent promising approaches for carefully selected patient populations. Nonetheless, clinical decision-making should continue to rely on robust evidence from well-designed clinical studies and individualized assessment of the potential risks and benefits of these interventions [50,51,57].

## Figures and Tables

**Table 1:** Studies assessing the associations between oxidative stress biomarkers and central nervous system alterations in major depressive disorder (MDD).

Author (Year)	Study Design	Scale Used	Population/Ethnicity	Exposure	Outcome	Methodological Evaluation
Liu et al. (2024) [45]	Case–control study.	Original Newcastle–Ottawa Scale (NOS).	A total of 57 adults with first-episode, drug-naïve MDD (18–55 years) and 60 healthy controls matched by age and sex. Ethnicity was not specified in the information provided.	Plasma superoxide dismutase (SOD) and glutathione reductase (GSR) levels. Resting-state functional magnetic resonance imaging (rs-fMRI) was performed to assess the amplitude of low-frequency fluctuations (ALFF) and functional connectivity.	SOD and GSR were associated with altered spontaneous activity in the thalamus, anterior cingulate cortex, and superior frontal gyrus. GSR showed a negative association with thalamus–insula connectivity, whereas SOD showed a positive association with thalamus–precuneus connectivity. The association patterns differed according to the presence or absence of suicidal ideation.	Selection: 3; comparability: 1; outcome/exposure: 2; total NOS score: 6. Classified as low risk of bias, according to the criteria applied in the review.
Peng et al. (2025) [46]	Case–control study.	Original Newcastle–Ottawa Scale (NOS).	A total of 44 Chinese patients with MDD and 47 healthy controls.	Plasma malondialdehyde (MDA) concentration and catalase (CAT) activity. Cognitive performance was assessed using the Repeatable Battery for the Assessment of Neuropsychological Status (RBANS) and the Stroop test.	Patients with MDD had higher MDA levels, lower CAT activity, and poorer performance in immediate memory, attention, delayed memory, and the RBANS total score. Higher MDA was associated with poorer mnemonic performance and greater depressive and anxiety symptom severity. Mediation analyses indicated that depression/anxiety severity may partially mediate the association between peripheral oxidative stress and cognitive performance.	Selection: 3; comparability: 2; outcome/exposure: 2; total NOS score: 7. Classified as low risk of bias.
Lee et al. (2025) [47]	Three-group case–control study: healthy controls vs. MDD vs. obsessive–compulsive disorder (OCD).	Original Newcastle–Ottawa Scale (NOS).	A total of 30 healthy controls, 28 patients with MDD, and 28 patients with OCD. Ethnicity was not specified in the information provided.	Cerebral glutathione (GSH) and glutamate quantified in the medial prefrontal cortex and precuneus using magnetic resonance spectroscopy with a MEGA-PRESS sequence; spontaneous neuronal activity was assessed using rs-fMRI.	GSH concentrations in the medial prefrontal cortex and precuneus were lower in the MDD and OCD groups than in healthy controls. Associations between GSH concentration and spontaneous neuronal activity metrics were explored; no association with clinical symptoms was reported.	Selection: 3; comparability: 1; outcome/exposure: 2; total NOS score: 6. Classified as low risk of bias.
Erbay et al. (2019) [43]	Pre–post interventional study.	National Heart, Lung, and Blood Institute (NHLBI) Quality Assessment Tool for Before–After (Pre–Post) Studies With No Control Group.	A total of 18 patients with MDD who received repetitive transcranial magnetic stimulation (rTMS). Ethnicity was not specified in the information provided.	rTMS intervention and prefrontal metabolites measured before and after treatment using proton magnetic resonance spectroscopy (1H-MRS), including N-acetylaspartate/creatine (NAA/Cr), GSH/Cr, and glutamine/creatine (Gln/Cr). Depression severity was measured with the Hamilton Depression Rating Scale (HAMD).	After rTMS, HAMD scores decreased significantly, and NAA/Cr, GSH/Cr, and Gln/Cr ratios increased significantly. These findings indicate concurrent clinical improvement and changes in the prefrontal neurochemical environment related to redox balance and glutamatergic neurotransmission.	The NHLBI assessment identified limitations in eligibility/selection criteria and absence of intervention blinding; the study was not an interrupted time-series design. The tool was regarded as providing guidance, rather than a standardized numerical scale, and described the overall included evidence as having moderate-to-high methodological quality.

Abbreviations: CAT, catalase; Gln, glutamine; GSH, glutathione; GSR, glutathione reductase; HAMD, Hamilton Depression Rating Scale; MDA, malondialdehyde; MDD, major depressive disorder; NOS, Newcastle–Ottawa Scale; OCD, obsessive–compulsive disorder; RBANS, Repeatable Battery for the Assessment of Neuropsychological Status; rs-fMRI, resting-state functional magnetic resonance imaging; rTMS, repetitive transcranial magnetic stimulation; SOD, superoxide dismutase. Note: Ethnicity and other characteristics not explicitly reported in the original source publications are indicated as “not specified” rather than inferred by the review authors.

## Data Availability

No new data were created; the data supporting this review are derived from previously published and properly cited studies. This review was registered in the PROSPERO database: CRD420261326016, https://www.crd.york.ac.uk/PROSPERO/view/CRD420261326016, accessed on 31 July 2026.

## Supplementary Materials

The following supporting information can be downloaded at: https://www.mdpi.com/article/10.3390/ijms27177961/s1.

## Abbreviations

The following abbreviations are used in this manuscript:

MDD	Major Depressive Disorder
ROS	Reactive Oxygen Species
RNS	Reactive Nitrogen Species
SOD	Superoxide Dismutase
GPx	Glutathione Peroxidase
CAT	Catalase
GSH	Reduced Glutathione
GSR	Glutathione Reductase
NO	Nitric Oxide
HPA	Hypothalamic–Pituitary–Adrenal Axis
BDNF	Brain-Derived Neurotrophic Factor
PRISMA	Preferred Reporting Items for Systematic Reviews and Meta-Analyses
MeSH	Medical Subject Headings
NOS	Newcastle–Ottawa Scale
JCR	Journal Citation Reports
rs-fMRI	Resting-State Functional Magnetic Resonance Imaging
fMRI	Functional Magnetic Resonance Imaging
MRS	Magnetic Resonance Spectroscopy
1H-MRS	Proton Magnetic Resonance Spectroscopy
rTMS	Repetitive Transcranial Magnetic Stimulation
ALFF	Amplitude of Low-Frequency Fluctuations
DTI	Diffusion Tensor Imaging
SVD	Small Vessel Disease
SUA	Serum Uric Acid
HAMD	Hamilton Depression Rating Scale
HAMD-24	24-Item Hamilton Depression Rating Scale
OCD	Obsessive–Compulsive Disorder
MDA	Malondialdehyde
RBANS	Repeatable Battery for the Assessment of Neuropsychological Status
CRP	C-Reactive Protein
