# Supplementary material for: Oxidative Stress and Neuroimaging Alterations in Major Depressive Disorder: A Systematic Review
Source: Int J Mol Sci. 2026 Sep 7;27(17):7961. doi: 10.3390/ijms27177961 (PMC13566424; doi:10.3390/ijms27177961)
Supplement: Supplementary file 1 [file ijms-27-07961-s001.zip › ijms-4459605-supplementary.pdf]

PRISMA 2020 Checklist

Section and Topic	Item #	Checklist item	Location where item is reported
**TITLE**			
Title	1	Identify the report as a systematic review.	Page 1, lines 2-3 (title)
**ABSTRACT**			
Abstract	2	See the PRISMA 2020 for Abstracts checklist.	Page 1, lines 19-33 (abstract)
**INTRODUCTION**			
Rationale	3	Describe the rationale for the review in the context of existing knowledge.	Introduction, p. 1-3, lines 39-180
Objectives	4	Provide an explicit statement of the objective(s) or question(s) the review addresses.	Abstract, p. 1, lines 22-26; Introduction, p. 3, lines 144-147
**METHODS**			
Eligibility criteria	5	Specify the inclusion and exclusion criteria for the review and how studies were grouped for the syntheses.	Section 2.2 Eligibility Criteria, p. 4, lines 19-210. Grouping for synthesis: section 2.5, p. 7, lines 280-284
Information sources	6	Specify all databases, registers, websites, organizations, reference lists and other sources searched or consulted to identify studies. Specify the date when each source was last searched or consulted.	P. 4, lines 191-197 (databases and search date: January 5, 2026); page 5, lines 214-215 (manual review of reference lists)
Search strategy	7	Present the full search strategies for all databases, registers and websites, including any filters and limits used.	P. 5, lines 214-228 (combined search terms and Boolean operators).
Selection process	8	Specify the methods used to decide whether a study met the inclusion criteria of the review, including how many reviewers screened each record and each report retrieved, whether they worked independently, and if applicable, details of automation tools used in the process.	P. 6, lines 229-233 (Figure 2 and footnotes). Note: the number of reviewers is not specified for screening, only for risk-of-bias assessment (item 11).
Data collection process	9	Specify the methods used to collect data from reports, including how many reviewers collected data from each report, whether they worked independently, any processes for obtaining or confirming data from study investigators, and if applicable, details of automation tools used in the process.	Section 2.3 Data Extraction, p. 6, lines 242-246. Number of reviewers and whether they worked independently are not specified.
Data items	10a	List and define all outcomes for which data were sought. Specify whether all results that were compatible with each outcome domain in each study were sought (e.g. for all measures, time points, analyses), and if not, the methods used to decide which results to collect.	P. 6, lines 245-246 (biomarkers, neuroimaging/neurofunctional measures, directionality and associations)
	10b	List and define all other variables for which data were sought (e.g. participant and intervention characteristics, funding sources).	P. 6, lines 242-246 (title,

PRISMA

PRISMA 2020 Checklist

Section and Topic	Item #	Checklist item	Location where item is reported
		Describe any assumptions made about any missing or unclear information.	design, population characteristics: ethnicity, age, sample size)
Study risk of bias assessment	11	Specify the methods used to assess risk of bias in the included studies, including details of the tool(s) used, how many reviewers assessed each study and whether they worked independently, and if applicable, details of automation tools used in the process.	Section 2.4 Risk of Bias Assessment Across Studies, p. 7, lines 248-276 (NOS and NHLBI tool)
Effect measures	12	Specify for each outcome the effect measure(s) (e.g. risk ratio, mean difference) used in the synthesis or presentation of results.	Not applicable – no pooled effect measures were calculated (no meta-analysis was performed; see p. 7, lines 280-281)
Synthesis methods	13a	Describe the processes used to decide which studies were eligible for each synthesis (e.g. tabulating the study intervention characteristics and comparing against the planned groups for each synthesis (item #5)).	P. 7, lines 280-284 (section 2.5 Synthesis of Results)
	13b	Describe any methods required to prepare the data for presentation or synthesis, such as handling of missing summary statistics, or data conversions.	Not explicitly reported
	13c	Describe any methods used to tabulate or visually display results of individual studies and syntheses.	Table 1, p. 6, lines 299-307; narrative synthesis, sections 3.1.1-3.1.4, p. 11-12, lines 320-377
	13d	Describe any methods used to synthesize results and provide a rationale for the choice(s). If meta-analysis was performed, describe the model(s), method(s) to identify the presence and extent of statistical heterogeneity, and software package(s) used.	P. 7, lines 280-284: narrative synthesis by biomarker type and neuroimaging modality; meta-analysis was not performed due to heterogeneity, p. 11, lines 317-334
	13e	Describe any methods used to explore possible causes of heterogeneity among study results (e.g. subgroup analysis, meta-regression).	Not applicable – no meta-analysis was performed
	13f	Describe any sensitivity analyses conducted to assess robustness of the synthesized results.	Not applicable – no meta-analysis was performed
Reporting bias assessment	14	Describe any methods used to assess risk of bias due to missing results in a synthesis (arising from reporting biases).	Not reported – methods to assess reporting bias/missing results are not described
Certainty assessment	15	Describe any methods used to assess certainty (or confidence) in the body of evidence for an outcome.	Not reported – no certainty assessment was performed (e.g., GRADE)
**RESULTS**			
Study selection	16a	Describe the results of the search and selection process, from the number of records identified in the search to the number of studies included in the review, ideally using a flow diagram.	P. 6, lines 229-238-203 (Figure 2, PRISMA flow diagram); p. 5, 6 lines 214-238 (descriptive text of the

PRISMA 2020 Checklist

Section and Topic	Item #	Checklist item	Location where item is reported
			process)
	16b	Cite studies that might appear to meet the inclusion criteria, but which were excluded, and explain why they were excluded.	P. 6, lines 230-236 (Figure 2 footnote: "Reason 1: Animal study", n=1). Only the general reason is given, without citing the specific excluded study.
Study characteristics	17	Cite each included study and present its characteristics.	Section 3.1 General Characteristics of the Included Studies, p. 11, lines 320-334; Table 1, p. 7,8, lines 229-307
Risk of bias in studies	18	Present assessments of risk of bias for each included study.	Table 1, p. 7,8, lines 229-307 (NOS, NHLBI assessment scores); text, p. 7, lines 247-276
Results of individual studies	19	For all outcomes, present, for each study: (a) summary statistics for each group (where appropriate) and (b) an effect estimate and its precision (e.g. confidence/credible interval), ideally using structured tables or plots.	Sections 3.1.1-3.1.4, p. 11-12, lines 335-377
Results of syntheses	20a	For each synthesis, briefly summarize the characteristics and risk of bias among contributing studies.	Table 1, p. 7,8, lines 229-307, risk of bias 2.4 section, lines 248-276.
	20b	Present results of all statistical syntheses conducted. If meta-analysis was done, present for each the summary estimate and its precision (e.g. confidence/credible interval) and measures of statistical heterogeneity. If comparing groups, describe the direction of the effect.	Discussion, p. 12, lines 380-407 (narrative synthesis; meta-analysis not applicable)
	20c	Present results of all investigations of possible causes of heterogeneity among study results.	Not applicable – no meta-analysis was performed
	20d	Present results of all sensitivity analyses conducted to assess the robustness of the synthesized results.	Not applicable – no meta-analysis was performed
Reporting biases	21	Present assessments of risk of bias due to missing results (arising from reporting biases) for each synthesis assessed.	Not reported
Certainty of evidence	22	Present assessments of certainty (or confidence) in the body of evidence for each outcome assessed.	Not reported
**DISCUSSION**			
Discussion	23a	Provide a general interpretation of the results in the context of other evidence.	Discussion, p. 12, lines 380-407 Conclusions, p. 15-16, lines 601-631
	23b	Discuss any limitations of the evidence included in the review.	Section 4.1 Limitations and Methodological Considerations, p. 15-16, lines 559-597
	23c	Discuss any limitations of the review processes used.	p. 15-16, lines 559-597Limitations specific to the review process itself (e.g.,

PRISMA 2020 Checklist

Section and Topic	Item #	Checklist item	Location where item is reported
			single reviewer for screening, only 3 databases) are not detailed separately.
	23d	Discuss implications of the results for practice, policy, and future research.	Conclusions, p. 15-16, lines 601-631
**OTHER INFORMATION**			
Registration and protocol	24a	Provide registration information for the review, including register name and registration number, or state that the review was not registered.	P. 17, lines 647-648 (Data Availability Statement): registration in PROSPERO: CRD420261326016.
	24b	Indicate where the review protocol can be accessed, or state that a protocol was not prepared.	Protocol Available from https://www.crd.york.ac.uk/PROSPERO/view/CRD420261326016.
	24c	Describe and explain any amendments to information provided at registration or in the protocol.	Not reported
Support	25	Describe sources of financial or non-financial support for the review, and the role of the funders or sponsors in the review.	P. 17, line 642 (Funding: "This research received no external funding)
Competing interests	26	Declare any competing interests of review authors.	P. 17, line 658 (Conflicts of Interest: "The authors declare no conflicts of interest.")
Availability of data, code and other materials	27	Report which of the following are publicly available and where they can be found: template data collection forms; data extracted from included studies; data used for all analyses; analytic code; any other materials used in the review.	P. 17, lines 645-648 (Data Availability Statement)

From: Page MJ, McKenzie JE, Bossuyt PM, Boutron I, Hoffmann TC, Mulrow CD, et al. The PRISMA 2020 statement: an updated guideline for reporting systematic reviews. BMJ 2021;372:n71. doi: 10.1136/bmj.n71.
This work is licensed under CC BY 4.0. To view a copy of this license, visit https://creativecommons.org/licenses/by/4.0/
